# Combined administration of laminin-221 and prostacyclin agonist enhances endogenous cardiac repair in an acute infarct rat heart

**DOI:** 10.1038/s41598-021-00918-y

**Published:** 2021-11-15

**Authors:** Nagako Sougawa, Shigeru Miyagawa, Takuji Kawamura, Ryohei Matsuura, Akima Harada, Yoshiki Sakai, Noriko Mochizuki-Oda, Ryoko Sato-Nishiuchi, Kiyotoshi Sekiguchi, Yoshiki Sawa

**Affiliations:** 1 Department of Cardiovascular Surgery, Osaka University Graduate School of Medicine, Suita, Osaka Japan; 2 Division of Matrixome Research and Application, Institute for Protein Research, Osaka University, Osaka, Japan

**Keywords:** Angiogenesis, Cardiac regeneration

## Abstract

Although endogenous cardiac repair by recruitment of stem cells may serve as a therapeutic approach to healing a damaged heart, how to effectively enhance the migration of stem cells to the damaged heart is unclear. Here, we examined whether the combined administration of prostacyclin agonist (ONO1301), a multiple-cytokine inducer, and stem cell niche laminin-221 (LM221), enhances regeneration through endogenous cardiac repair. We administered ONO1301- and LM221-immersed sheets, LM221-immersed sheets, ONO1301-immersed sheets, and PBS-immersed sheets (control) to an acute infarction rat model. Four weeks later, cardiac function, histology, and cytokine expression were analysed. The combined administration of LM221 and ONO1301 upregulated angiogenic and chemotactic factors in the myocardium after 4 weeks and enhanced the accumulation of ILB4 positive cells, SMA positive cells, and platelet-derived growth factor receptor alpha (PDGFRα) and CD90 double-positive cells, leading to the generation of mature microvascular networks. Interstitial fibrosis reduced and functional recovery was prominent in LM221- and ONO1301-administrated hearts as compared with those in ONO1301-administrated or control hearts. LM221 and ONO1301 combination enhanced recruitment of PDGFRα and CD90 double-positive cells, maturation of vessels, and functional recovery in rat acute myocardial infarction hearts, highlighting a new promising acellular approach for the failed heart.

## Introduction

Heart failure, including myocardial infarction, is often progressive and continues to be a major factor contributing to morbidity and mortality worldwide^1^. Recently developed treatment strategies such as cell-based or drug therapy may potentially regenerate diseased heart^2^. However, the application of cell therapy is limited owing to the need for cell culture process, unsuitability with emergency use, and low versatility. Drugs that enhance the endogenous healing process may potentially regenerate the damaged heart tissue without the need for cell culture process and avoid the shortcomings associated with cell therapy. Therefore, induction of endogenous healing to enhance the recruitment of stem cells is thought as a promising approach to regenerate the damaged heart tissue. And also, how to effectively enhance the migration of stem cells to the damaged heart can be crucial for endogenous tissue repair.

ONO1301, a synthetic prostacyclin agonist with thromboxane A2 synthase inhibitory activity, has been shown to act as a multiple-cytokine inducer by ligating prostaglandin I (IP) receptors expressed on endothelial cells, vascular smooth muscle cells (vSMCs), or fibroblasts. Our laboratory demonstrated that administration of ONO1301 for acute and chronic ischemic cardiac failure animal models yielded proangiogenic cytokines, such as vascular endothelial growth factor (VEGF) or hepatocyte growth factor (HGF), and stromal cell-derived factor-1 (SDF-1), contributing to the repair of the tissues and inducing functional recovery^3–6^.

The extracellular matrix (ECM) is essential for the structural support, maintenance, and differentiation of tissues^7^ and proposes a microenvironment that is suitable to support stem cell proliferation, migration, and fate decision^8^. In particular, basement membranes contain structural proteins, including laminins, that are bound to the plasma membrane via specific receptors such as integrins^9^. Laminin-221 (LM221) is the most likely expressed cardiac laminin that maintains physiological activities of myocytes^9,10^. In addition, previously, we demonstrated that in rat acute myocardial infarction (AMI) model, administration of LM221 enhanced accumulation of smooth muscle actin (SMA) and isolectin B4 (ILB4) double-positive cells to the damaged area, and contributed to the improvement of cardiac function^11^.

In this study, we hypothesized that the combined treatment with a prostacyclin agonist (ONO1301) and stem cell niche LM221 may enhance regeneration by mediating endogenous cardiac repair.

## Results

### Combined administration of LM221 and ONO1301 enhanced cytokine expression

**Figure 1:**
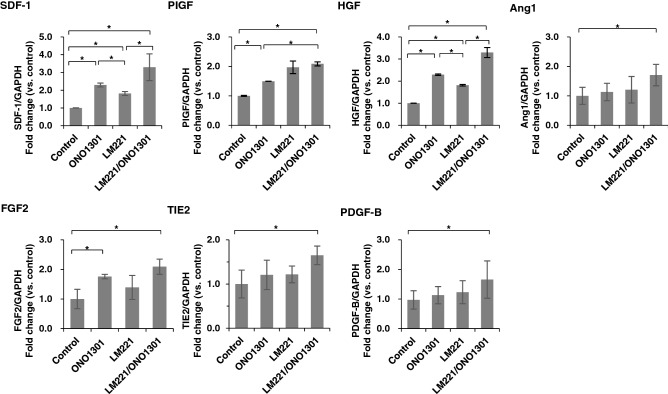
Combined administration of LM221 and ONO1301 induced chemotactic cytokine and angiogenic cytokine gene expression. The expression of *SDF-1*, *PlGF*, *HGF*, *Ang1*, *FGF2*, *TIE2*, and *PDGF-B* in the infarcted area was measured by quantitative reverse-transcription polymerase chain reaction. Data were normalised to *GAPDH* expression. *p < 0.05.

We first analysed the expression of cytokines 4 weeks after treatment. We found that the expression of the cytokines related to angiogenesis and cell migration changed in the border area (Fig. 1). The relative gene expression levels of chemotactic factor such as *SDF-1* was induced a significant elevation in LM221- and ONO1301-administration hearts compared to other groups (Control hearts; 1.00 ± 0.02, ONO1301-administrated hearts; 2.29 ± 0.11, LM221-administrated hearts; 1.81 ± 0.11, LM221- and ONO1301-administrated hearts; 3.29 ± 0.75, *p* < 0.05). In addition, combined administration of LM221 and ONO1301 in AMI model hearts led to increased gene expression of angiogenic factors such as *HGF*, placental growth factor (*PlGF*), angiopoietin 1 (*Ang1*), fibroblast growth factor 2 (*FGF2*), *Tie2*, and platelet-derived growth factor beta (*PDGF-B*). The relative expression of PIGF was remarkably enhanced in LM221- and ONO1301-administrated hearts compared to control hearts and ONO1301-administrated hearts (Control hearts; 1.00 ± 0.06, ONO1301-administrated hearts; 1.49 ± 0.002, LM221- and ONO1301-administrated hearts; 2.09 ± 0.07, *p* < 0.05). However, there was no significant difference between LM221- and ONO1301-administrated hearts and LM221-administrated hearts. Although the relative expression of HGF was remarkably enhanced in LM221- and ONO1301-administrated hearts compared to control hearts and LM221-administrated hearts (Control hearts; 1.00 ± 0.02, LM221-administrated hearts; 1.81 ± 0.04, LM221- and ONO1301-administrated hearts; 3.30 ± 0.23, *p* < 0.05), there was no significant difference between LM221- and ONO1301-administrated hearts and ONO1301-administrated hearts. On the other hand, significant enhancement of gene expression of Ang1, FGF2, Tie2, and PDGF-B in LM221- and ONO1301-administrated hearts were observed only compared with control hearts.

**Figure 2:**
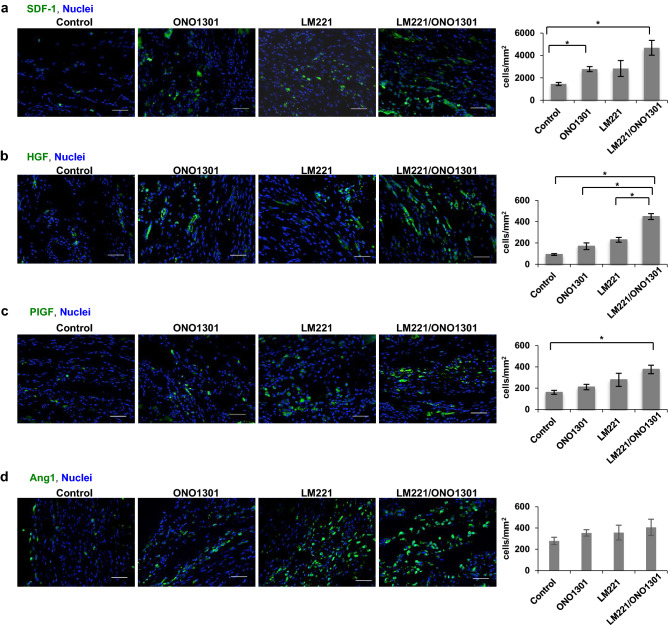
Combined administration of LM221 and ONO1301 induced chemotactic cytokine and angiogenic cytokine production. The protein expression of (**a**) SDF-1, (**b**) HGF, (**c**) PIGF, and (**d**) Ang1 in the implanted site was measured by immunohistochemistry. Representative microscopic images show cytokines (green) and nuclei (blue). Scale bar = 50 μm. The graph indicates the number of cytokine-positive cells at the implanted site. *p < 0.05.

The protein expression of these cytokines in LM221- and ONO1301-administrated hearts was also significantly higher than that in other hearts (Fig. 2). Four weeks after treatment, the number of SDF-1-positive cells in LM221- and ONO1301-administrated hearts were 4689.3 ± 662.1 cells/mm^2^, which was significantly higher than that in control hearts (1453.3 ± 134.3 cells/mm^2^, *p* < 0.05) (Fig. 2a), and, that of PlGF-positive cells in LM221- and ONO1301-administrated hearts was significantly higher than that in control hearts (160.5 ± 18.6 cells/mm^2^, *p* < 0.05) (Fig. 2c). Similarly, the number of HGF-positive cells in LM221- and ONO1301-administrated hearts was 447.7 ± 27.7 cells/mm^2^, which was significantly higher than that in control (91.0 ± 8.2 cells/mm^2^, *p* < 0.05), ONO1301- (169.1 ± 31.6 cells/mm^2^, *p* < 0.05), and LM221-administrated hearts (228.9 ± 22.5 cells/mm^2^, *p* < 0.05). On the other hand, the number of Ang1-positive cells in LM221- and ONO1301-administrated hearts was not a significant elevation compared to other hearts (Fig. 2d).

### Combined administration of LM221 and ONO1301 suppressed cell apoptosis

**Figure 3:**
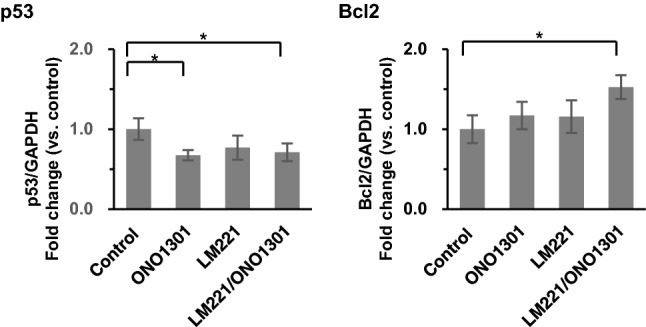
Combined administration of LM221 and ONO1301 decreased cell apoptosis. The expression of *p53*, *Bcl2* in the infarcted area was measured by quantitative reverse-transcription polymerase chain reaction. Data were normalised to *GAPDH* expression. *p < 0.05.

To examine the effects of administration on cell death, we analysed apoptotic factors. The relative expression levels of *p53* gene in ONO1301-administrated, and LM221- and ONO1301-administrated hearts were 0.67 ± 0.06, and 0.71 ± 0.11, respectively, which were significantly lower than that in control hearts (*p* < 0.05). On the other hand, the relative expression levels of *Bcl2* gene in LM221- and ONO1301-administrated hearts was which was significantly higher than that in control hearts (*p* < 0.05) (Fig. 3).

### Combined administration of LM221 and ONO1301 promoted capillary maturation

**Figure 4:**
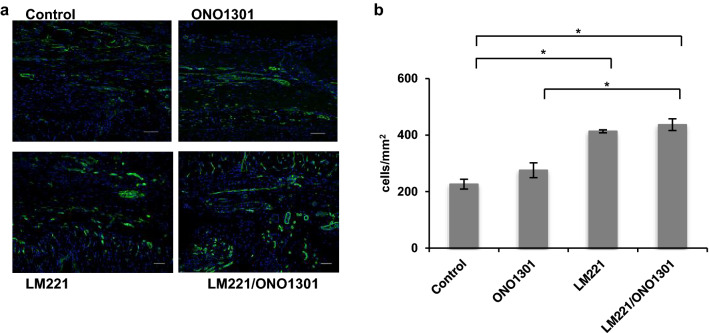
Combined administration of LM221 and ONO1301 increased ILB4-positive cells. The number of ILB4-positive cells in the implanted site increased following combined administration of LM221 and ONO1301, as observed by immunohistochemical analysis. Representative microscopic images show ILB4 (green) and nuclei (blue). Scale bar = 50 μm. The graph indicates the number of ILB4-positive cells at the implanted site. *p < 0.05.

**Figure 5:**
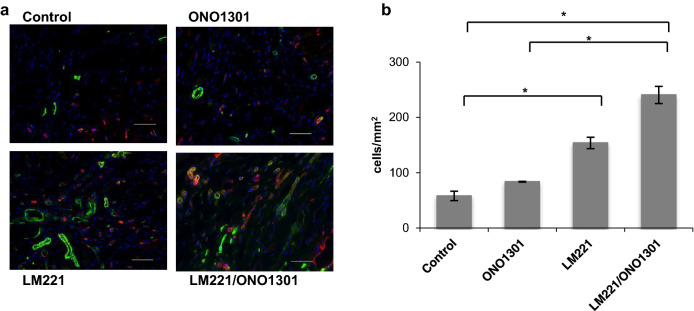
Combined administration of LM221 and ONO1301 increased mature capillary density. Some ILB4-positive cells in the implanted site were coated with SMA-positive cells, as observed by immunohistochemical analysis. ILB4 and SMA double-positive cells increased following the combined administration of LM221 and ONO1301. Representative microscopic images show ILB4 (red), SMA (green), and nuclei (blue). Scale bar = 50 μm. The graph indicates the number of ILB4 and SMA double-positive cells at the implanted site. *p < 0.05.

Next, we investigated the nature of the cells that accumulated at the damaged site. Isolectin B4 (ILB4)-positive cells accumulated in the border area. The number of ILB4-positive cells in LM221- and ONO1301-administrated hearts (436.9 ± 20.8 cells/mm^2^) was significantly higher than that in control hearts (226.3 ± 17.3 cells/mm^2^, *p* < 0.05) and ONO1301-administrated hearts (275.6 ± 26.4 cells/mm^2^, *p* < 0.05). However, those in LM221- and ONO1301-administrated hearts and LM221-administrated hearts (413.4 ± 5.4 cells/mm^2^) was almost same (Fig. 4). On the other hand, the number of mature capillaries^12,13^ indicated with ILB4-positive vessels^14^ undercoated with SMA-positive cell in LM221- and ONO1301-administrated hearts (240.6 ± 15.5 cells/mm^2^) tended to be higher than that in LM221-administrated hearts (153.8 ± 10.4 cells/mm^2^ cells/mm^2^). In addition, the number of mature capillaries in LM221- and ONO1301-administrated hearts significantly increased compared to that in control hearts (57.9 ± 8.4 cells/mm^2^, *p* < 0.05) and ONO1301-administrated hearts (83.7 ± 0.7 cells/mm^2^, *p* < 0.05) (Fig. 5).

### Combined administration of LM221 and ONO1301 increased PDGFRα and CD90 double-positive cell accumulation

**Figure 6:**
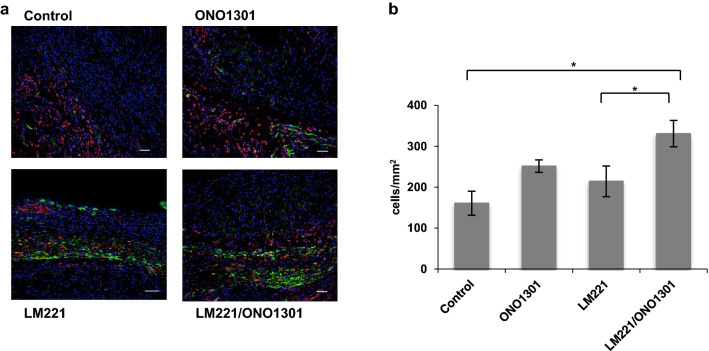
Combined administration of LM221 and ONO1301 increased *PDGFRα and CD90 double-positive cell accumulation.* PDGFRα and CD90 double-positive cells increased after the combined administration of LM221 and ONO1301, as observed by immunohistochemical analysis. Representative microscopic images show PDGFRα (red), CD90 (green), and nuclei (blue). Scale bar = 50 μm. The graph indicates the number of PDGFRα and CD90 double-positive cells at the implanted site. *p < 0.05.

The number of PDGFRα and CD90 double-positive cells in LM221- and ONO1301-administrated hearts (330.9 ± 32.2 cells/mm^2^) was significantly higher than that in control hearts (160.8 ± 19.3 cells/mm^2^, *p* < 0.05) and LM221-administrated hearts (214.5 ± 37.5 cells/mm^2^, *p* < 0.05), however, that didn’t show a significant increase compared to that in ONO1301-administrated hearts (251.5 ± 15.1 cells/mm^2^) (Fig. 6).

### Combined administration of LM221 and ONO1301 drove the infarct size suppression

**Figure 7:**
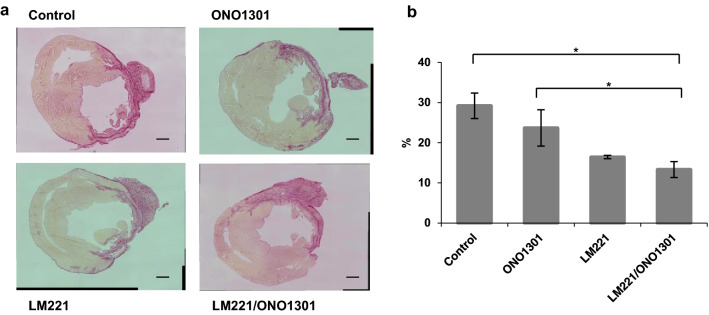
The combined administration of LM221 and ONO1301 reduced fibrosis. Fibrosis was observed by Picro-Sirius Red staining. (**a**) Representative microscopic images of the infarct size. Scale bars = 1000 μm. (**b**) The graph indicates the size of fibrosis at the implanted site 4 weeks after surgery. *p < 0.05.

The size of infarcted area in LM221- and ONO1301-administrated hearts (13.3% ± 2.0%) was significantly suppressed that of in control hearts (29.2% ± 3.2%, *p* < 0.05) and in ONO1301-administrated hearts (23.7% ± 4.5%, *p* < 0.05), while there was no significant difference between that of in LM221-administrated hearts (16.4% ± 0.4%) and LM221- and ONO1301-administrated hearts (Fig. 7).

### Combined administration of LM221 and ONO1301 ameliorated the cardiac function

**Figure 8:**
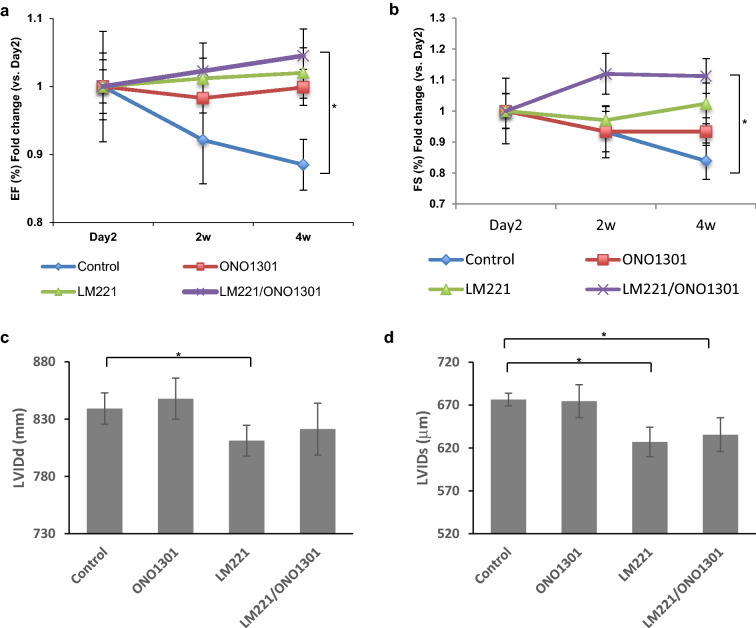
The combined administration of LM221 and ONO1301 improved heart performance. (**a**,**b**) Line graphs depicting the left ventricle ejection fraction (EF, **a**) and fractional shortening (FS, **b**) 2 days and 2 and 4 weeks after treatment. (**c**,**d**) The statistical analysis of the echocardiographic results of the diastolic (LVIDd, **c**) and systolic (LVIDs, **d**) left ventricular internal diameter 4 weeks after treatment. *p < 0.05.

To assess the pathological effect, we evaluated the cardiac function in each group. As shown in Fig. 8a,b, control hearts showed a time-dependent decrease in the percentage of ejection fraction (EF) and fractional shortening (FS). ONO1301-administrated hearts and LM221-administrated hearts didn’t show much fluctuation in the percentage of EF and FS. However, the combined treatment with LM221 and ONO1301 displayed a time-dependent increase in the percentage of EF and FS. The relative EFs and FSs for LM221- and ONO1301-administrated hearts were significantly higher than that for control hearts 4 weeks after treatment (*p* < 0.05). However, there were no significant differences among LM221- and ONO1301-administrated hearts, LM221-administrated hearts, and ONO1301-administrated hearts (Fig. 8a,b). Although the left ventricle internal dimensions at diastole (LVIDd) in LM221- and ONO1301-administrated hearts was not a significant improvement, the left ventricle internal dimensions at systole (LVIDs) in the LM221- and ONO1301-administrated hearts was significantly improved compared to control hearts 4 weeks after treatment (Fig. 8c,d).

## Discussion

Although cell-based therapy is a promising strategy for heart failure treatment, it is limited owing to the need for cell culture process, unsuitability with emergency use, and low versatility. To overcome these issues, we suggested a new acellular strategy in this study. Direct epicardial placement of LM221 and ONO1301 over the cardiac surface upregulated the expression of angiogenic and chemotactic factors in the myocardium at 4 weeks after transplantation. In addition, the combined administration of LM221 and ONO1301 suppressed cell death, and enhanced the accumulation of PDGFRα and CD90 double-positive cells or endothelial cells, leading to the generation of microvascular networks accompanied by SMA-positive cells. Lower fibrosis and effective functional recovery were shown in LM22-1 and ONO1301-administrated hearts than in ONO1301-administrated hearts, LM221 treated hearts and control hearts.

The vascular system is stabilized by the action of pericytes/vSMCs, a process termed as maturation. Vascular maturation has been reported to be dependent on the members of the vascular endothelial growth factor (VEGF) family, FGF2, PDGF-B, and Ang1. The VEGF family or FGF2 play a critical role in the expression and recruitment of endothelial cells^15^. PDGF-B secreted by endothelial cells controls mural cell recruitment to the endothelial cells^16^. Ang1 produced by mural cells activates endothelial cells and maximizes their interaction with mural cells^16^. These cytokine-related angiogenic processes produce a mature vascular system that avoids serious leakage and hemorrhage^15,16^. ONO1301 activates endothelial cells, vascular smooth muscle cells and fibroblasts to release multiple cytokines^3^. On the other hand, LM221 acts as a scaffold to store cells, growth factors and cytokines^7^. In the present study, we confirmed the significant upregulation in the expression of chemotactic factors and angiogenic factors (SDF-1, HGF, FGF2, PIGF, PDGF-B, and Ang1) after treatment with the combination of LM221 and ONO1301, suggesting that this combined strategy enhances vascular maturation mediated by cytokine cocktails. Although the expression of VEGF was not significantly different among the groups (data not shown), that of PlGF, a member of the VEGF family, was significantly upregulated by the administration of ONO1301, and LM221 and ONO1301 compared to the control. Thus, PlGF may contribute to the sprouting of the vasculature instead of VEGF. In addition, HGF, FGF2, and PlGF, which were induced by the combined administration of LM221 and ONO1301, enhanced the proliferation and recruitment of ILB4-positive endothelial cells, while PDGF-B promoted vSMC migration to the vicinity of endothelial cells. Ang1 subsequently facilitated endothelial cell coverage via vSMCs^16^. We speculate that vascular maturation in our model may be mediated by the mechanism described above.

The newly formed blood vessels through the action of a single cytokine may not have sufficient interaction with the surrounding vascular network and are not covered with mural cells. Vessels without mural cells demonstrate immature characteristics, leading to vascular leakage, hemorrhage, and early regression^15^. In this study, the combined administration of LM221 and ONO1301 significantly increased the number of structurally mature vessels, as evident from ILB4-positive endothelial cells accompanied with SMA-positive cells as compared to the other groups. Thus, the combined strategy mediated effective angiogenesis for tissue repair. Although the origin of SMCs is not clearly elucidated in our experimental model, another cell type such as CD11b^+^ cells with c-kit expression or Sca-1^+^ cells having α7 integrin (a receptor for LM221) may have potentially differentiated into SMCs^7,17^. The accumulation of Sca-1^+^ cells at the border area was observed both in LM221-administrated and LM221- and ONO1301-administrated hearts (data not shown). And that made us speculate that the possibility of Sca-1^+^ cells differentiating into mural cells at the transplanted site.

The administration of LM221 alone was insufficient to accumulate PDGFRα and CD90 double-positive cells as compared with the combined treatment because these cells cannot be retained at the treatment site owing to the low expression of α7 integrin. These data suggest that SDF-1 induced by ONO1301 enhanced PDGFRα and CD90 double-positive cell recruitment into the transplanted site. In addition, the production of cytokines and the number of accumulated cells were higher in LM221- and ONO1301-administrated hearts than in the other hearts, suggesting the possibility that the cytokine expression was derived from endothelial cells activated by recruited cells.

Tissue homeostasis and wound repair are ensured by stem cells located within specialised microenvironments, referred to as niches. The stem cell niche comprises stem cells, neighboring cells, ECM, and secreted factors that are essential for cell survival and proliferation as well as to maintain the stability of undifferentiated stem cells^18^. The ECM is a scaffold for cells containing adhesion molecules such as integrins and is critical for the maintenance of the cytoskeleton as well as cell survival, growth, and fate^19^. LM221 is most abundantly expressed in the adult cardiac muscle and specifically binds to α7 integrin expressed in the myocardium^9,20^. LM221 is a scaffold for myocardiocytes and can influence cell maturation and survival through outside-in signaling^21^. These data suggest that the combined administration of LM221 and ONO1301 in vivo promotes stem cell differentiation, proliferation, and consequently improves cardiac function.

**Figure 9:**
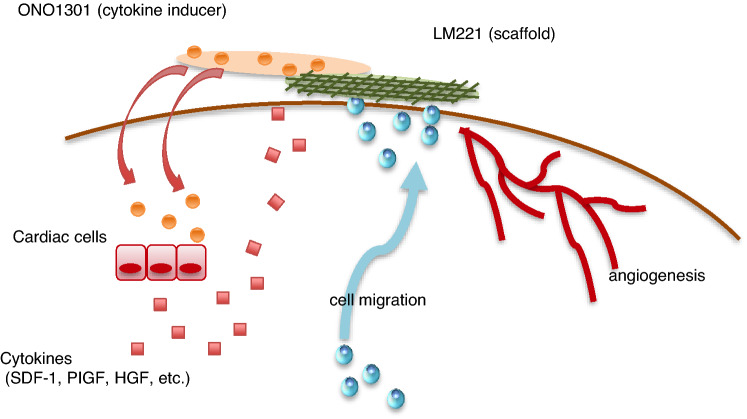
Schematic figure of proposed mechanisms underlying LM221 and ONO1301-administrated treatment of myocardial infarction heart. ONO1301 is linearly released and infiltrated into the cardiac tissue. IP receptor expressing cardiac cells, such as vascular smooth muscle cells, fibroblast and endothelial cells, are activated by binding of ONO1301, leading to paracrine released cytokines, such as SDF-1, PIGF, or HGF. Cells, such as PDGFRα and CD90 double-positive cell, endothelial cell, or smooth muscle cell, are locally induced by SDF-1, and cells accumulate in the place using LM221 as a scaffold, leading to promotion of angiogenesis.

We proposed the following mechanisms in this paper. ONO1301 is linearly released and infiltrated into the cardiac tissue. IP receptor expressing cardiac cells, such as vascular smooth muscle cells, fibroblasts and endothelial cells, are activated by binding of ONO1301, leading to paracrine released cytokines, such as SDF-1, PIGF, or HGF. Cells, such as PDGFRα and CD90 double-positive cell, endothelial cell, or smooth muscle cell, are locally induced by SDF-1, and cells accumulate in the place using LM221 as a scaffold, leading to promotion of angiogenesis and recovering the cardiac function (Fig. 9). Totally, although the administration of ONO1301 alone or LM221 alone was insufficient in improvement in AMI model hearts, the combined administration with ONO1301 and LM221 was significantly more effective at suppression of infarct size and improvement of cardiac function. In conclusion, direct epicardial placement of LM221 and ONO301 promoted recruitment of PDGFRα and CD90 double-positive cells, maturation of vessels, and functional recovery in rat acute MI hearts, suggesting the promising role of this acellular approach for failed hearts.

## Materials and methods

### Animal care

Animal care was performed as previously described^11,22^. The animal care procedures used in this study was reviewed and proved by the Guide for the Care and Use of Laboratory Animals (National Institutes of Health publication no. 85-23, revised 1996 no. 85-23, revised 1996). The experimental protocols were approved by the Ethics Review Committee for Animal Experimentation of Osaka University Graduate School of Medicine (reference number 28-011-002) and adhered to the ARRIVE guidelines. All surgeries and sacrifices were performed under deep anesthesia with isoflurane sufficient to minimize animal suffering. All experiment procedures and evaluations were performed in a blinded manner.

### Recombinant LM

Human LM221 was produced as a recombinant functionally active E8 fragment containing the collagen-binding domain of fibronectin at the N-terminus to facilitate binding to collagen sheets^23–25^. Recombinant LM221 was expressed using a FreeStyle 293 Expression System (Life Technologies, Carlsbad, CA, USA) and purified by sequential affinity chromatography using Ni–NTA agarose (Qiagen, Carlsbad, CA, USA) and anti-FLAG-M2-agarose (Sigma, St. Louis, MO, USA)^16^.

### Ligation of the left coronary artery and epicardial placement of collagen sheet in rats

LM221 was resuspended in phosphate-buffered saline (PBS) at 100 nM concentration and applied to atelocollagen sheets (KOKEN, Tokyo, Japan) through immersion for 2 h at 37 °C. ONO1301 (Ono Pharmaceutical Co. Ltd., Osaka, Japan) was resuspended in saline and applied to atelocollagen sheets. Male athymic nude rats aged 6–8 weeks (F344$\cdot$NJcl-*rnu/rnu*) were obtained from CLEA Japan (Tokyo, Japan). Left thoracotomy was performed under general anesthesia via the fourth intercostal space, and the branch of the left coronary artery was permanently ligated at the bottom of the left atrial appendage. Five minutes after ligation, LM221- and ONO1301-immersed atelocollagen sheets (KOKEN, Tokyo, Japan) (n = 8), LM221-immersed atelocollagen sheets (n = 10), ONO1301-immersed atelocollagen sheets (n = 10), or atelocollagen sheets (n = 11) were directly placed over the anterolateral surface of the left ventricular (LV) wall. After the approximation of the surgical wound, the rats were maintained in a temperature-controlled individual cage and then euthanized 4 weeks after the surgical procedure.

### Quantitative reverse transcription polymerase chain reaction (PCR)

Quantitative reverse transcription polymerase chain reactions were performed as previously described^11^. Total RNA was extracted from excised cardiac tissues and reverse transcribed using SuperScript III Reverse Transcriptase (Invitrogen) with random primers (Invitrogen). Quantitative PCR was performed using a ViiA7 Real-Time PCR System (Applied Biosystems, Carlsbad, CA, USA) with rat-specific primers (Applied Biosystems) for PGF (Assay ID: Rn00585926_m1), FGF2 (Assay ID: Rn00570809_m1), HGF (Assay ID: Rn00566673_m1), and SDF-1 (Assay ID: Rn01462855_m1), and glyceraldehyde-3-phosphate dehydrogenase (GAPDH) (Assay ID: Rn01775763_g1). The specific primer sets for Ang1, TIE2, PDGF-B, Caspase-3, p53, Bcl2 were as follows: Ang1, 5ʹ-gag cat aaa atc cta gaa atg gag gg-3ʹ and 5ʹ-tgc aga aca atg ttg ttg ctg gta gc-3ʹ; TIE2, 5ʹ-cag gac ctt cac aac agc ttc tat cgg act-3ʹ and 5ʹ-ctg tcg aag aat gtc act aag ggt cca agc-3ʹ; PDGF-B, 5ʹ-gct cct ttg atg acc ttc agc-3ʹ and 5ʹ-cag ccc gag cag cgc tgc acc tc-3ʹ; p53, 5ʹ-ccc agg gag tgc aaa gag ag-3ʹ and 5ʹ-tct cgg aac atc tcg aag cg-3ʹ; Bcl2, 5ʹ-cct gtg gat gac tga gta cct-3ʹ and 5ʹ-gag cag ggt ctt cag aga ca-3ʹ; GAPDH, 5ʹ-ctc aag ggc tgt ggg caa ggt cat-3ʹ and 5ʹ-gag atc cac cac cct gtt gct gta-3ʹ. Each sample was analysed in triplicates for each gene. Data were normalised to GAPDH expression. For relative expression analysis, the delta-delta Ct method was used, and the values for the group treated with atelocollagen sheet only were used as reference.

### Histological analysis

Histological analyses were performed as previously described ^11^. The collected hearts were fixed with 4% paraformaldehyde (PFA) and embedded in optimal cutting temperature compound for sectioning. Frozen Sects. (5 μm) of transverse heart slices were incubated with antibodies against PDGFRα, CD90, SDF-1, HGF, PGF, Ang1 (Abcam, Cambridge, UK) and SMA (Dako, Glostrup, Denmark). Samples were then probed with Alexa Fluor 555- or 488-conjugated secondary antibodies (Invitrogen, Carlsbad, CA, USA). The frozen sections were also labeled with AlexaFluor 647-conjugated ILB4 (Invitrogen) following the manufacturer’s instructions. Nuclei were counterstained with Hoechst 33,342 (Dojindo, Kumamoto, Japan). The labeled sections were assessed using a Biorevo BZ-9000 (Keyence, Osaka, Japan). Finally, 5–10 fields were captured for each specimen, and the results were expressed as cell density. Transversely sliced heart sections were stained with Picro-Sirius Red and assessed by optical microscopy. Percent fibrosis was calculated as the percentage of pink-colored collagen in the total area.

### Transthoracic echocardiography

Transthoracic echocardiography was performed as previously described ^11^. Cardiac function was assessed using an echocardiography system with a 12-MHz transducer (Sonos 7500; Philips, Andover, MA, USA) 2 days, 2 weeks, and 4 weeks after MI. Standard transthoracic echocardiography was performed under anesthesia with isoflurane inhalation. The left ventricular end-diastolic and end-systolic diameters (LVEDD and LVESD, respectively) were measured, whereas the left ventricular end-diastolic and end-systolic volumes (LVEDV and LVESV, respectively) were calculated using the Teichholz formula. The left ventricular ejection fraction (LVEF) was calculated using the following formula: LVEF (%) = 100 × (LVEDV − LVESV)/(LVEDV). The left ventricular fraction shortening (LVFS) was calculated using the following formula: LVFS (%) = 100 × (LVEDD − LVESD)/(LVEDD).

### Statistical analysis

Data are expressed as mean ± standard error of the mean. Data distributions were checked for normality. Multiple comparisons were assessed using one-way analysis of variance (ANOVA) with Tukey–Kramer post-hoc testing. All *p* values were two-sided, and values of *p* < 0.05 were considered statistically significant.

## Abbreviations

AMI: Acute myocardial infarction
Ang1: Angiopoietin 1
ECM: Extracellular matrix
EF: Ejection fraction
FGF2: Fibroblast growth factor 2
FS: Fractional shortening
GAPDH: Glyceraldehyde-3-phosphate dehydrogenase
HGF: Hepatocyte growth factor
ILB4: Isolectin B4
IP: Prostaglandin I
LM: Laminin
LVIDd: Left ventricle internal dimensions at diastole
LVIDs: Left ventricle internal dimensions at systole
LVEDV: Left ventricular end-diastolic volume
LVESV: Left ventricular end-systolic volume
MSC: Mesenchymal stem cell
PCR: Polymerase chain reaction
PDGFRα: Platelet-derived growth factor receptor α
PDGF-B: Platelet-derived growth factor beta
PlGF: Placental growth factor
SDF-1: Stromal cell-derived factor-1
SMC: Smooth muscle cell
SMA: Smooth muscle actin
VEGF: Vascular endothelial growth factor
vSMC: Vascular smooth muscle cell
